# Profuse nocturnal eyelid haemorrhage from a spider angioma

**DOI:** 10.1111/aos.70080

**Published:** 2026-01-24

**Authors:** Ype P. de Jong, Paulus T. V. M. de Jong

**Affiliations:** ^1^ Division of Gastroenterology and Hepatology Weill Cornell Medicine New York New York USA; ^2^ Department of Ophthalmology AmsterdamUMC Amsterdam The Netherlands; ^3^ Department of Retinal Signal Processing Netherlands Institute of Neuroscience, Royal Academy of Arts and Sciences Amsterdam The Netherlands

**FIGURE 1:**
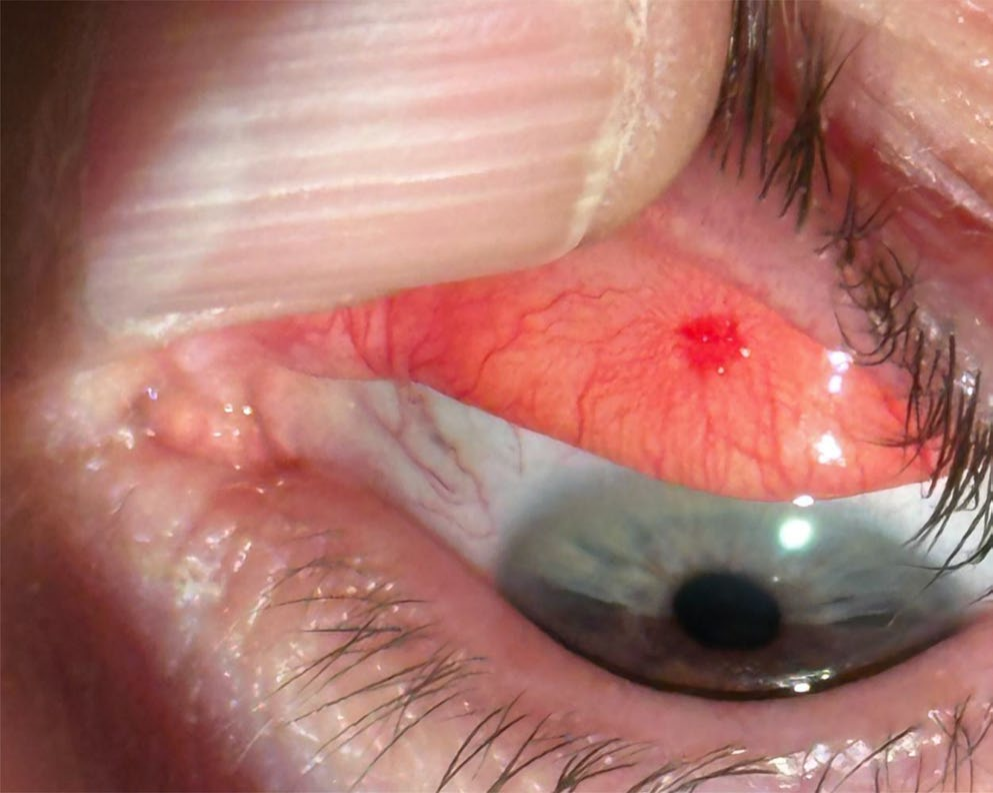
Spider angioma in the left upper tarsal conjunctiva which caused twice in 2 years a massive nocturnal haemorrhage. Photo taken 3 days after its last bleeding episode.

At 2:45 a.m., a 47‐year‐old Caucasian woman awoke unable to open her left eye due to a large blood clot covering her eyelid. After washing away the clot and changing the blood‐soaked pillowcase, she resumed sleep. A similar episode of nocturnal bleeding had occurred 1 year prior. The patient wore disposable contact lenses and was certain she had removed them before going to bed. She was healthy, taking 40 mg of pantoprazole daily and denied the use of anticoagulants or medication affecting platelet function. She had used oral contraceptives for 30 years, discontinuing them 2 years earlier. Three days after the second bleeding episode, the patient's eye examination revealed visual acuity 1.0 OU with Sph −10.0D. Slit‐lamp examination showed no trace of blood, inflammation or redness on the ocular surface, conjunctiva, or in the anterior chamber OU, and otherwise, both eyes only revealed an optic disc consistent with her myopia. Upon everting the left upper eyelid, a spider angioma (also known as spider naevus or spider telangiectasia) was observed on the tarsal conjunctiva (Figure 1). Her husband later conducted a full skin check, revealing a single additional spider angioma on her left upper leg.

Spider angiomas on the skin were present in 47.5% of healthy children between the ages of 3 and 17 years, with a peak prevalence between 7 and 10 years (Alderson, 1963). In adults, they are common in pregnancy and in patients with cirrhosis of the liver. In the latter, they are mostly located on the upper thorax, neck, face and in mucous membranes of the nose, mouth, pharynx, hard and soft palate and in the linings of the alimentary tract (Bean, 1953). Extensive literature searches in PubMed, Medline and ophthalmological reference works revealed no mention of a spider angioma in the tarsal conjunctiva. Stewart Duke‐Elder described in his System of Ophthalmology conjunctival spider‐like telangiectasias only after ionizing radiation treatment. Last year, a spider telangiectasia was described on both bulbar conjunctivae of a 45‐year‐old male with cirrhosis of the liver (Chavan & Rajput, 2025).

Spider angiomas are generally regarded as a benign and transient finding during normal pregnancy, with a prevalence of 60%–70% among white and approximately 10% among black women (Henry et al., 2006). The association between oral contraceptive use and the development of spider naevi has been only sparsely documented (Deharo et al., 1988). In liver cirrhosis, spider naevi are attributed to impaired hepatic metabolism and clearance of androstenedione, resulting in increased extrahepatic conversion to oestrogen. Our patient demonstrated no evidence of advanced liver disease (Fibrosis Index Based‐4 score: 0.88). We therefore consider her long‐term use of oral contraceptives to be the most likely etiologic factor in the development of her spider angiomas. Mechanical trauma from her contact lens wear or removal may have led to rupture of the angioma wall. The patient declined treatment due to concern about potential conjunctival scarring, and no further episodes of bleeding have occurred.
